# Encouragement of American Scientific Labors

**Published:** 1853-02

**Authors:** 


### EDITORIAL DEPARTMENT.

---

*Encouragement of American Scientific Labors.*—We have repeatedly taken occasion to comment on the tendency, in this country, to undervalue the labors of our own countrymen in behalf of medical science. Foreign works and communications in the Medical Journals of other countries, are reprinted, copied, circulated and absorb the attention, to the neglect of native productions which, to say the least, are equally meritorious, and which therefore, it would be supposed, should claim at least a fair relative consideration on the score of nationality. This disposition to depreciate everything of American origin, and to magnify everything emanating abroad, is by no means confined to medical literature. It pervades all the operations of mind, from the inventive genius which produces the never-ending variations in the fashion of dress, to the most important matters connected with scientific truth. A coat or boot made after the latest mode at the metropolis of the new Empire, is the *beau ideal* of excellence, and so the latest lucubrations of some unknown German, French, or English writer, is almost sure to be greeted with the utmost cordiality, while the truly creditable efforts of our fellow countrymen are but too often unnoticed. We do not find fault with any stretch of courtesy. or generosity, toward the labors of our brethren in other lands, all being fellow-artizans in the great republic of science which should, be limited by no territorial or sectional boundaries; still less do be desire to see a degree of national assumption, or pride of patriotism, which might conflict with a recognition of superior claims whenever they are justly due to developments, scientific or otherwise, which come to us across the Atlantic. What we object to is, that undue importance should be attached to what is foreign simply because it is so, and on the other too little estimation placed upon native productions in consequence of their domestic origin. We complain, too, of the habit of thinking that the ore from our own scientific mines, must pass through a foreign mint before it is entitled to currency at home. It has happened more than once that practical improvements have been communicated on this side of the water which have been scarcely noticed till they attracted the attention of a journalist or author in Europe, and thus endorsed, they succeeded in obtaining a recognition in our own country. How often is it said with emphasis that such or such an one has acquired an "*European reputation.*" This, we admit, is legitimately enough a matter of congratulation, or pride; but it is far from being complimentary to ourselves to present the fact as a reason why a person should enjoy with us a better character than before. It is customary to grumble at the disparaging remarks sometimes made by foreign writers respecting American contributions to the general stock of intellectual acquirement. We smart under a sense of injustice conveyed in the sneers in which it pleases some illnatured scribbler of another nation, occasionally to indulge. But this we fairly deserve so long as we virtually admit the charge of inferiority by a want of proper appreciation and encouragement of the labors of our countrymen. If we do not evince self-respect, we have no right to expect that marked consideration will be accorded to us by others.

Our thoughts have been directed to this subject by reading an editorial article by our friend, the editor of the Southern Medical and Surgical Journal. At the conclusion of a volume of his excellent periodical, he says: "Thirty-two writers, residing in nine different states, have furnished us fifty-two original articles for the last volume, which will, we think, compare favorably with the original department of any of our contemporaries. The great majority of these papers are essentially practical, very few of them being theoretical disquisitions. This we regard as an important feature in this publication, and have always borne it in mind in making up the eclectic department. Among our selections, we count sixty-six original *American* articles, derived from our respected exchanges, thus making no less than one hundred and eighteen contributions to medical knowledge by our countrymen, in the volume for 1852! We are not of those who will transfer to their columns every idle lucubration or hasty suggestion found in foreign prints, while they pass without notice the writings, however valuable, of their fellow-citizens. This indifference, manifested by some of our contemporaries, to the dissemination of American contributions, has long been felt and complained of by the profession — and while we know that we have not republished as many of them as we could have wished, we have the satisfaction to reflect that we have done as much as our limits would permit. There are Abstracts, Retrospects, and Periscopes published in Europe, republished in the United States, and bought extensively by our professional brethren, and which yet scarcely contain an allusion to any thing written or done in this country. Why so? Can it be that nothing is done or written by the American medical profession worthy of a place in such compilations? Dr. Norwood, and others, have now for two years been publishing, from time to time, in this Journal, the discovery and usefulness of an agent capable of controlling with almost mathematical certainty, the action of the heart in disease. There are upward of thirty medical periodicals in the United States, with all of which, save one, we exchange. Of these, we doubt whether more than five or six have even noticed the discovery, directly or indirectly. How different would the case have been if the newly ascertained property of veratrum viride had been first announced in England, in France, or even in the depths of Germany! We might cite similar instances of disregard of valuable contributions published in the Journals of almost every section of our country."

After the remarks introductory to the quotation just made, it is needless to say that we honor the views and conduct of the writer, and they must commend themselves to the approbation of every truly *American* reader.

Since the foregoing was written, we have happened to meet, in another of our contemporaries, statements very similar to those which we have expressed. In noticing the late treatise on Operative Surgery, by Henry H. Smith, M.D., contained in the Phil. Med. Examiner, for Jan., we are glad to observe that the editor comments, with proper severity, on the custom of deriving materials for systematic works from foreign authors, to the neglect of the abundant resources existing at home. We quote the following: "The materials for a genuine cis-Atlantic work may yet be had abundantly within the reach of those amongst us — and there should be many such — who are competent and not afraid to use them. The task, however discouraging it may now appear, must sooner or later be fulfilled, without this resort to the enervating draughts from foreign sources, which of late years have been the cause of so many of our publications. No man need go to Paris or London now to learn how to amputate a limb or extirpate a tumor, reset a bone, extract a calculus — in short, to effect the greatest triumph which surgical art and science, under Providence, have ever yet achieved. Why, then, this never-ending slavery to the parasitic feebleness which will scarcely allow us, without resorting to an unknown tongue, to show our pupils how to perform the simplest operation."

Politically, the United States of America hold an independent position, and are fast becoming the most conspicuous and powerful of the nations of the world. Let the American mind be fully emancipated, and it is destined to accomplish for science, literature and art, results commensurate with those which pertain to civil and religious freedom!

